# Influence of water washing treatment on *Ulva prolifera*-derived biochar properties and sorption characteristics of ofloxacin

**DOI:** 10.1038/s41598-021-81314-4

**Published:** 2021-01-19

**Authors:** Chenghu Yang, Shichao Miao, Tiejun Li

**Affiliations:** 1 Key Laboratory of Sustainable Utilization of Technology Research for Fishery Resource of Zhejiang Province, Zhejiang Marine Fisheries Research Institute, Zhoushan, 316021 Zhejiang People’s Republic of China; 2 Marine and Fishery Institute, Zhejiang Ocean University, Zhoushan, 316021 People’s Republic of China

**Keywords:** Environmental sciences, Ocean sciences

## Abstract

The influences of water washing treatment on the properties of *Ulva prolifera*-derived biochar (*U.P-*biochar) and its sorption characteristics of ofloxacin (OFL) were investigated. The results showed that the water washing treatment significantly changed the physiochemical structures of *U.P-*biochars, and improved the sorption capacity of OFL. The sorption capacity of OFL by *U.P*-biochar was closely dependent on pyrolysis temperature (200–600 °C) and equilibrium solution pH (3–11). Different sorption mechanisms (e.g. cation exchange, electrostatic attraction, H-bond and cationic–π and π–π interactions) were dominant for specific *U.P-*biochars under various pH regions (acidic, neutral and alkaline). Moreover, the unwashed and washed *U.P-*biochars prepared at 200 °C (BC200 and BCW200) showed a higher sorption capacity of OFL at pH = 7. The two-compartment first-order model provided an appropriate description of the sorption kinetics of OFL by BC200 and BCW200 (*R*^2^ > 0.98), which revealed that the contribution ratios between the fast and slow sorption compartments (*f*_fast_*/f*_slow_, 1.55 for BC200 and 1.25 for BCW200) reduced after water washing treatment of *U.P*-biochar. The values of n for the Freundlich model were less than 1, which demonstrated that the sorption of OFL by BC200 and BCW200 was favourable and nonlinear. Also, the sorption of OFL by BC200 and BCW200 increased with an increase in solution temperature and the sorption process was spontaneous and endothermic. This study provides valuable information for being a primary consideration in the production and application of *U.P-*biochar.

## Introduction

Antibiotics have received intensive attention for the treatment and prevention of infectious diseases in humans and livestock, especially in high-intensity animal rearing and aquaculture^1^. Meanwhile, antibiotics are indirectly or directly introduced into water and soil through human and animal excreta, untreated wastewater or the discharge of aquaculture products^2^. These actions have resulted in the development and spread of antibiotic resistance, concomitantly causing adverse effects on the ecosystem and human health^3,4^. Statistically, more than 700,000 deaths per year have resulted from antimicrobial resistance since 2014 and it is expected to rise continually to 10 million deaths by 2050^5^. Consequently, the management of antibiotics in various environments has drawn great attention.

Numerous methods have been developed to remove antibiotics from water, including sorption, chemical oxidation, photochemical degradation, biological degradation and electrochemical degradation^6^. Among these treatment techniques, sorption has consistently been a superior method because of its relatively low cost, high efficiency and simplicity of operation^7,8^. Besides, sorption is a necessary process controlling the fate and bioavailability of antibiotics in the environment^9,10^. Biochar, a black carbon-rich material obtained from the pyrolysis of organic biomassunder oxygen-limited conditions, has been prevalently recognised as a readily available and environmentally friendly sorbent for contaminant management in water and soil^11,12^. Previous investigations have indicated that biochar could be potentially applied for the sorption of antibiotics from waste water by the combination of multiple mechanisms (e.g. electrostatic attraction, π–π electron-donor–acceptor interaction and hydrogen bond (H-bond) and hydrophobic interactions)^7,13^.

It is confirmed that the sorption effects of pollutants by biochar are closely related to its physicochemical properties (e.g. surface area, high cation exchange capacity and surface functional groups), which significantly depend on the biomass source and pyrolysis conditions^14,15^. Recently, macroalgae have been used as the precursor for preparing biochars because of their massive abundance and easily acquired nature^16^. Moreover, the conversion of biomass to biochar as one potential end-use will improve the resource utilisation of macroalgae, especially for the ‘pest’ species (e.g. green tide algae) that exhibit rapid growth and environmental tolerance in eutrophic environments^17^. *Ulva prolifera* (syn. *Enteromorpha prolifera*)^18^, a dominant green-tide-forming macroalgae, is distributed diffusely in the intertidal zones of coastal areas around the world^19^. Since its first major occurrence in 2008, the green-tide-forming phenomenon has become an annual event during early summer in the Yellow Sea, causing serious environmental concerns for coastal cities^20^. Prior reports have detailed that biochar produced from *U. prolifera* successfully exhibited strong sorption capacities for heavy metal, dyes and organic contaminants^21,22^. However, limited information on the sorption characteristics between antibiotics and *U. prolifera*-derived biochar (*U.P*-biochar), is available.

Also, the water-soluble materials of biochar, including inorganicchemicals and dissolved organic matter (DOM), would be released once itcontacts aquatic environments. This process may change thesurface properties and porous structures of biochar and subsequently affect its sorption behaviour for contaminants^23,24^. Notably, both positive and negative influences of water washing process on the sorption capacity of biochar have been observed in the diverse reaction systems. For instance, Gai et al. demonstrated that additional sorption sites on the biochar surface might be created to facilitate the sorption of more nitrate N by removing ash from biochars with water washing treatment^25^. However, Boakye et al. showed that the sorption capacity of *Saccharina japonica*-derived biochar for crystal violet decreased after water washing, resulting from a decrease in the cation exchange between biochar and dye^26^. Therefore, it is critical to further focus on the roles of water washing treatment on the sorption of contaminants on biochar, which may provide a strategy for the production and potential application of biochar.

The solution pH is considered one of the main influencing factors in the sorption of antibiotics in water because of the charge distribution on the biochar surface and the ionisation states of antibiotics were different at various pH ranges. Previous studies showed that the effects of initial pH on the sorption of antibiotics by biochar were dependent on pyrolysis temperature, attributing to the multiple roles that pH plays^7^. However, the initial solution pH might be observably changed in the presence of biochar, which was incapable of indicating the influences of practical pH values on the sorption between biochar and antibiotics^27,28^. Therefore, understanding the equilibrium pH-dependence of biochar’s sorption is crucial for developing efficient remediation approaches.

Ofloxacin (OFL), as one of the major antibiotics consumed in China, has been frequently detected in various environmental media and was chosen as a model antibiotic compound^29^. The primary goal of this work was to evaluate the differences in the properties of washed and unwashed *U.P*-biochars and those sorption behaviours of OFL, including the pyrolysis temperature and equilibrium solution pH effects. Meanwhile, multiple sorption mechanisms were discussed based on the structural effects of *U.P*-biochar and OFL. In addition, the sorption kinetics, sorption isotherms, and thermodynamic analysis were performed to further investigate the sorption characteristics of OFL by *U.P*-biochar.

## Results and discussion

### General properties of U.P-biochar

Table 1 lists the elementary composition and atomic ratio of *U.P-*biochar before and after the water washing treatment. First, significant differences in the elemental contents and atomic ratios were observed between *U. prolifera* and its derived biochar, clarifying that biomass properties were changed with limited-oxygen pyrolysis. On the whole, there was no obvious variation in the C content, while the N, O and H contents for *U.P-*biochar decreased with increasing pyrolysis temperature. The atomic ratio of H/C decreased with the pyrolysis temperature, suggesting that the aromaticity of *U.P-*biochar at a low pyrolysis temperature was significantly lower than that at a high pyrolysis temperature^30^. The decreasing O/C ratio indicated an increase in the carbonisation degree and hydrophobicity and the decreased polarity index [(O + N)/C] reflected the reduction of O-containing polar functional groups with increasing pyrolysis temperature^31^. Furthermore, the C, H and N contents increased, while the O content remained stable with the water washing treatment, primarily due tothe ash and DOM of pristine *U.P-*biochar being removed^22^. The water washing treatment decreased the aromaticity and amount of O-containing functional groups in *U.P-*biochar according to the higher H/C ratio and lower [(O + N)/C] ratio. By contrast, the washed *U.P-*biochar was a highly carbonised and hydrophobic structure, which was reflected by a lower O/C ratio.

**Table 1:** Physiochemical characteristics of *U. prolifera* and *U.P-*biochars.

Biochars	C (%)	N (%)	O (%)	H (%)	H/C	O/C	(O + N)/C	pH_pzc_	BET surface area (m^2^/g)	Total pore volume (cm^3^/g)	Average pore size (nm)
BC0	31.42	2.539	37.56	5.122	1.956	0.897	0.966	6.60	3.22	0.0164	20.45
BC200	36.31	3.538	23.24	2.463	0.814	0.480	0.564	8.18	4.65	0.0050	4.27
BC300	37.95	3.541	21.40	2.587	0.818	0.423	0.503	8.50	19.04	0.0143	3.00
BC400	34.31	2.989	21.25	0.742	0.260	0.465	0.539	10.4	4.94	0.0904	73.21
BC500	33.65	2.618	18.53	0.631	0.225	0.413	0.480	10.9	6.06	0.0091	6.00
BC600	32.92	2.227	14.99	0.474	0.173	0.342	0.399	11.2	13.46	0.0067	2.33
BCW200	61.82	5.749	23.54	5.282	1.025	0.286	0.365	6.25	4.88	0.0189	15.52
BCW300	64.37	5.820	21.02	5.039	0.939	0.245	0.322	6.68	26.02	0.0582	8.95
BCW400	65.82	5.999	20.45	2.606	0.475	0.233	0.311	7.15	31.72	0.0685	8.64
BCW500	61.94	4.785	19.16	2.127	0.412	0.232	0.298	10.4	135.53	0.1920	5.66
BCW600	61.93	4.141	15.96	1.604	0.311	0.193	0.251	10.8	257.41	0.2830	4.40

The point of zero charge (pH_pzc_) significantly influences the sorption properties and sorption capacities between biochar and organic pollutants, especially ionizable organic compounds^32^. The pH_pzc_ values ranged from 8.18 to 11.2 and 6.25 to 10.81 for the pristine and washed *U.P-*biochars, respectively (Table 1). The pH_pzc_ generally increases with increasing pyrolysis temperature, demonstrating that *U.P-*biochar obtained at a higher pyrolysis temperature has a strongly basic surface. Meanwhile, the water washing treatment reduced the pH_pzc_ of *U.P-*biochar, suggesting the net surface charges of the pristine and washed biochar were different at the specific pH^33^. When the solution pH is lower than pH_pzc_, the surface of biochar is positively charged, resulting in a superior ability for the sorption of anionic species. Whereas when the solution pH is higher than pH_pzc_, the biochar surface tends to be negatively charged, leading to an enhancement in cationic species’ sorption ability^34^.

The BET surface area of washed *U.P-*biochars was larger than unwashed *U.P-*biochars (Table 1), which meaning that more sorption sites of *U.P-*biochars could be produced with water washing. Furthermore, the BET surface area of washed *U.P-*biochars increased with increasing pyrolysis temperature. The similar observations had been reported in previous publications^35,36^.

### FTIR spectra

The FTIR spectra of pristine and washed *U.P-*biochars are illustrated in Supplementary Information Fig. S1. The wide band near 3407 cm^−1^ was ascribed to the stretching of O–H in carboxyl and phenol functional groups or the N–H symmetric stretching vibration^22^. The characteristic peaks at approximately 2925 cm^−1^ and 2885 cm^−1^ were attributed to C–H stretching bands associated with aliphatic functional groups. The peak at around 1617 cm^−1^ was related to aromatic C=C or C=O, while the prominent peaks at approximately 1437 cm^−1^, 1139 cm^−1^ and 875 cm^−1^ were assigned to the stretches of C–H_2_, aliphatic C–O–C and aromatic C–H^25^. All the FTIR peak intensities of the pristine *U.P-*biochars exhibited a lower magnitude at a higher pyrolysis temperature, suggesting a decrease in the surface functional groups with an increasing pyrolysis temperature. In addition, significant differences were distinctly observed in the FTIR spectra of the washed and unwashed biochars, confirming that water washing effectively affects the functional groups of *U.P-*biochar. These bands presented different changes for specific *U.P*-biochars after the water washing treatment, and the variation trends of *U.P*-biochars with different pyrolysis temperatures were not completely similar. For instance, all the FTIR peak intensities of BCW200 were lower than those of BC200 except for the peak at approximately 2925 cm^−1^. A similar variation was also noted between BC300 and BCW300. After the water washing treatment, the peak at 1139 cm^−1^ became strongly diminished for the *U. prolifera*-derived biochars prepared at 400 °C and 500 °C, while other peaks were preserved. Compared with BC600, water washing decreased the intensities of surface functional groups assigned to 1139 cm^−1^, while three new structures (2925 cm^−1^, 1437 cm^−1^ and 882 cm^−1^) were formed in BCW600.

### Effects of pyrolysis temperature and pH on the sorption of OFL by the washed and unwashed U.P-biochars

**Figure 1:**
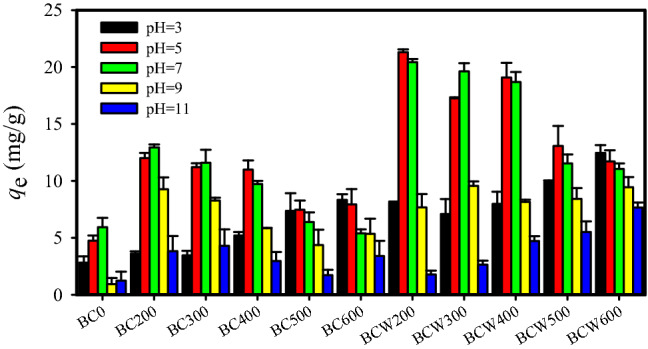
Effects of the pyrolysis temperature and pH on sorption of OFL by *U.P*-biochars. [Conditions] 20 mL of 20 mg/L OFL; 0.0150 g of *U.P*-biochar; 7 days. The experiment was conducted in triplicate.

As shown in Fig. 1, the sorption capacity of *U.P-*biochar was evidently higher than that of *U. prolifera*, suggesting that limited-oxygen pyrolysis ofbiomass could improve the affinity for OFL. Moreover, the sorption capacities of *U.P-*biochars for OFL were significantly dependent on the pyrolysis temperature and water washing treatment, which were attributed to the different properties of biochars with various preparation processes. Obviously, the sorption capacity of OFL by the washed *U.P*-biochar was greater than that by the unwashed *U.P*-biochar for a specific situation, revealing that the removal of ash and DOM by water washing generated more sorption sites on the washed *U.P*-biochar surface. Interestingly, the effects of pyrolysis temperature on *U.P-*biochars sorption of OFL were dependent on the solution pH. The sorption abilities of OFL by both washed and unwashed *U.P-*biochars increased with the pyrolysis temperature at pH 3, while the opposite trend was observed at pH 5 and 7. The unwashed *U.P-*biochar sorption capacity of OFL continuously decreased with increasing pyrolysis temperature in the range of 200 to 500 °C and increased at a pyrolysis temperature of 600 °C at pH 9, while the sorption capacity of washed *U.P-*biochar gradually increased with the pyrolysis temperature at pH 11. No apparent regularity was observed for the washed *U.P-*biochars sorption capacity at pH 9 and the unwashed *U.P-*biochars at pH 11 for a pyrolysis temperature ranged between 200 and 600 °C. Huang et al. previously stated that the sorption capacity of biochar for OFL increased with the pyrolysis temperature due to the biochars prepared at higher temperatures had more sorption sites^7^. However, another study revealed that the pyrolysis temperature was negatively correlated with the sorption capacity of OFL on biochar, because of the lower oxygen-containing functional groups and the organic distribution phase on the surface of biochar with higher pyrolysis temperature^37^. The aforementioned conflicting findings may be due to the fact that the properties of both the sorbents and the sorbate were different under their respective experimental conditions.

The various influences of pH on the sorption of OFL by *U.P-*biochar were also clearly observed. Accordingly, the washed and unwashed *U.P-*biochars displayed similar pH dependences of sorption for OFL in each series of *U.P-*biochars. Thus, we primarily focused on the effects of pH on the washed *U.P-*biochars. In detail, with the increase in pH, the sorption capacities of OFL on BCW200, BCW300, BCW400 and BCW500 increased at the beginning and reached a maximal value at asolution pH of approximately 5 or 7 and then decreased rapidly. It was noted that the sorption capacity of BCW600 continually decreased when the pH varied 3 to 11. These variation tendencies in a pH-dependent fashion were mainly due to the fact that the pH would strikingly shift the surface charge properties of biochar and the species distribution of OFL (OFL^+^, OFL^0^and OFL^−^)^38^. However, the present literature has reported that the sorption of OFL by biochars expressed no significant change for an initial pH ranging from 3 to 9^7^. The following reasons mainly cause the controversy regarding the effects of pH on the biochars sorption of OFL: (i) the characteristics of biochar from each biomass were unique, resulting in various sorption mechanisms for a further diverse tolerance to pH. (ii) The initial solution pH could be shifted due to the buffering effect of acidic (carboxyl) and basic (amine) functional groups as well as the alkaline inorganics on the surface of biochars and the process was dependent on various biochars^27^.

### Sorption mechanisms

The mechanisms of the above phenomena are discussed for in-depth understanding of the interaction between OFL and *U.P-*biochars and the washed *U.P-*biochars were selected as the representatives for this section. The discontinuous variation in the sorption capacity of *U.P-*biochars suggested that the sorption mechanisms involved in disparate pH regions were different. Given this, acidic (pH = 3 and 5), neutral (pH = 7) and alkaline (pH = 9 and 11) were roughly labelled as three various pH regions.

#### Acidic region

The solution pH (3 and 5) is lower than the pH_pzc_ of *U.P-*biochars (6.25–10.8) and the p*K*_a1_ (6.10) of OFL, resulting the surface of both sorbent and sorbate are positively charged. Thus, electrostatic repulsion occurred between OFL and the surface of *U.P-*biochars due to their like charges underacidic conditions^34^. Additionally, the electrostatic repulsion increased with the decrease in pH and the increase in pyrolysis temperature in this pH range. However, the substantially higher-than-expected sorption of cationic OFL suggested that cation exchange may have played a key role in the sorption of OFL by *U.P-*biochars. The sites for cation exchange may have been the oxygen-containing functional groups (such as carboxyl groups) on biochars^39^. A higher temperature could cause less diversity of functional groups on the surface of *U.P*-biochar, which may in turn affect the ionic exchange during sorption processes. Therefore, the sorption capacity of *U.P-*biochars would decline theoretically, with an increase in the pyrolysis temperature. Nevertheless, the contrary result was distinctly observed at pH 3, demonstrating that other mechanisms such as cationic–π and π–π interactions also participated in the sorption process^40^. The *U.P-*biochars formed at a higher pyrolysis temperature contained more aromatic structures, which may have improved the cationic–π and π–π interactions between biochars and cationic OFL.

#### Neutral region

The content of OFL^+^ decreased to 10.7%, but the OFL^0^ increased to 84.9% at a pH of 7 (Supplementary Information Fig. S2). Thus, the sorption capacity might reduce through the weakening ionic exchange and cationic–π interactions between biochar and OFL. However, the sorption capacity of *U.P-*biochars also exhibited a relatively high level at neutrality, which was probably due to H-bond, π–π and hydrophobic interactions being involved in the sorption process^38^. Meanwhile, the positive surface charge of *U.P-*biochars decreased due to the deprotonation effect, leading to significant reduction in the electrostatic repulsion between *U.P-*biochars and OFL. In addition, according to the characteristicsof *U.P-*biochars, the contribution of H-bond for biochars decreased with the pyrolysis temperature, while the contribution of π–π interactions increased with the pyrolysis temperature. Here, the OFL had a good hydrophilicity (i.e. log*K*_ow_ =  − 0.39) and the highest sorption values did not all appear at the lowest solubility, which suggested that the sorption of OFL by *U.P-*biochars was not entirely dependent on the hydrophobic interaction^38,39^.

#### Alkaline region

The π–π interaction was also involved in the sorption of OFL by *U.P-*biochars in the range of pH at 9–11, which increased with the pyrolysis temperature. The species OFL^−^ is the dominant form in this situation, which accounted for 84.0% and 98.1% at pH 9 and 11, respectively. The electrostatic repulsion was maintained between OFL and *U.P-*biochars obtained at a pyrolysis temperature ≤ 400 °C, while the electrostatic attraction was generated between OFL and *U.P-*biochars obtained at a pyrolysis temperature ≥ 500 °C, because these two types of *U.P-*biochars were negatively charged (≤ 400 °C) and positively charged (≥ 500 °C) at pH 9^34^. The negative charge-assisted H-bond (( − )CAHB) may serve as another important mechanism for the sorption of the anionic species of a hydrophilic compoundon a negatively charged carbon surface, which was easily generated as the H-bond donor and H-bond acceptor had comparable *pK*_a_ values^38^. The difference between the *pK*_a2_ (8.28) of OFL and the *pK*_a_ (strong carboxylic acid (*pK*_a_ 5–6.4), moderate acid/lactone (*pK*_a_ 6.4–10.3) and weak phenolic acid (*pK*_a_ 10.3–13))^41^ of *U.P-*biochars generated at a pyrolysis temperature ≤ 400 °C was less than 4, which may have been beneficial to produce (−)CAHB at pH 9. A *U.P-*biochar produced at alower pyrolysis temperature had a higher potential for (−)CAHB due to the abundance of its O-containing groups. Thus, the *U.P-*biochars showed similar sorption capacities due to the joint effects of the electrostatic attraction, electrostatic repulsion, π–π interaction and (−)CAHB at pH 9. The (−)CAHB would be significantly inhibited under strong alkaline conditions due to the excess of hydroxide ions^38^, leading to the sorption capacity of *U.P-*biochars for OFL being dramatically decreased at pH 11. In addition, both OFL and *U.P-*biochars became progressively negatively charged with the increase in pH, resulting in the electrostatic repulsion beingsignificantly improved at pH 11. As a consequence, the sorption capacities of *U.P-*biochars were further reduced under the stronger alkaline condition.

Additionally, the reasons for the promotion of sorption performance of OFL by *U.P-*biochars after washing treatment could be inferred to these various sorption mechanisms. Firstly, the aromaticity and amount of O-containing functional groups in *U.P-*biochar reduced after water washing treatment, which showed that the DOM in *U.P-*biochars contained aromatic substance and abundant functional groups. Thus, the DOM may bind with OFL through cation exchange, π–π interactions and H-bond, resulting in the competitive sorption with biochar for OFL in water. Secondly, the pH_pzc_ of unwashed *U.P-*biochar was higher than that of washed *U.P-*biochar, leading to a stronger electrostatic repulsion between OFL and biochar at specific pH (except pH 9).

### Characterisation of BC200 and BCW200 sorption for OFL

Above results showed that the *U.P-*biochar produced at apyrolysis temperature of 200 °C had greater sorption capacity of OFL at pH 7. Thus, BC200 and BCW200 were chosen to further investigate the sorption characteristics of *U.P-*biochar and OFL by the kinetics, isotherms, thermodynamics at neutrality.

#### Sorption kinetics

**Figure 2:**
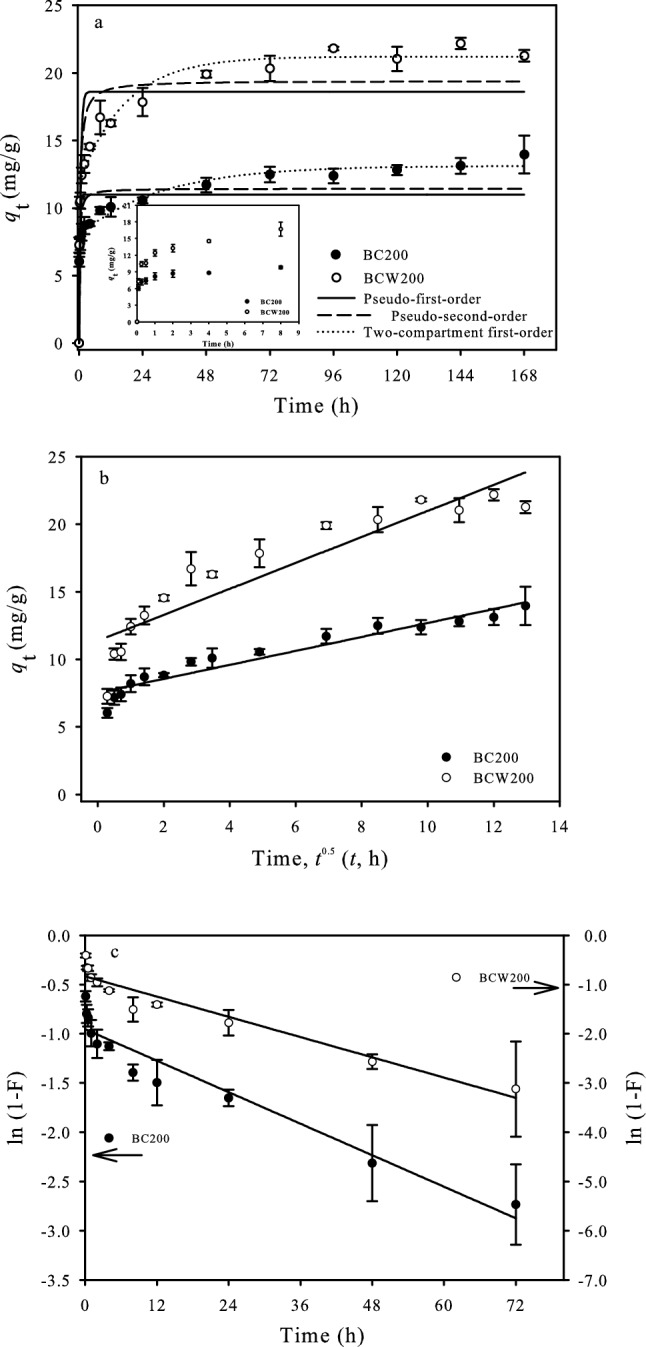
Sorption kinetics of OFL on *U. P-*biocharsby fitting the pseudo-first-order model, pseudo-second-order model and two-compartment first-order model (**a**) intraparticle diffusion model (**b**) and liquid-film diffusion model (**c**). [Conditions] 20 mL of 20 mg/L OFL; 0.0150 g of *U.P*-biochar; pH of 7.0 ± 0.2; 25 °C. The experiment was conducted in triplicate.

The sorption kinetics is popularly utilised to evaluate the sorption process and sorption mechanism. The sorption kinetic curves of OFL on BC200 and BCW200 showed that the sorption capacity of OFL on *U.P-*biochar increased with the contact time (Fig. 2a). The dynamic sorption trends of OFL on BC200 and BCW200 could be characterised by three stages including a rapid sorption within the initial 24 h, then a slow sorption, and finally an equilibrium stage at approximately 96 h. This may have been due to the abundance of readily accessible sorption sites on the surfaces of BC200 and BCW200 at the early stage and then the available active sites gradually declined with sorption time, resulting in the slower sorption rate^42,43^. The fitting parameters of the three different kinetic models are presented in Table 2. The pseudo-first-orderand pseudo-second-order models were not suitable to describe the sorption kinetics between OFL and *U.P-*biochars because of the relatively low correlation coefficients (*R*^2^, 0.708–0.865), which were not consistent with previously obtained results^44,45^. However, the two-compartment first-order model was considered as being the well-fitted model with thehighest correlation coefficients (*R*^2^ > 0.98). Previous investigations have also reported that the two-compartment first-order model could be employed precisely to explain the sorption of antibiotics (i.e. OFL and tetracycline) on biochar as a two-domain process^37,42^. The values of *k*_fast_ and *f*_fast_ were higher than those of *k*_slow_ and *f*_slow_, revealing that the fast sorption stage was the principal process in our study. The contribution of the fast sorption compartment to the sorption of OFL on BC200 was considerably greater than that on BCW200, which likely accounted for the O-containing functional groups of BC200 being higher than that of BCW200. The interaction between OFL and *U.P-*biochar is thought to occur via H-bonds and π–π interactions at neutrality, which may be the possible mechanisms for the rapid sorption of OFL^15^. The water washing process could decrease the contribution ratios between the fast and slow sorption compartments (*f*_fast_*/f*_slow_), which was possibly attributed to the removal of ash/minerals. It has been reported that the minerals of biochars played dominant roles in the fast sorption of organic compounds^46,47^. Correspondingly, the values of *k*_slow_ and *f*_slow_ of BCW200 were larger than those of BC200, which might have been dependent on the pore volume and size of BCW200 being higher than those of BC200 (0.0050 cm^3^/g and 4.27 nm for BC200, and 0.0189 cm^3^/g and 15.52 nm for BCW200). Furthermore, the calculated values of *q*_e,cal_ (13.13 mg/g for BC200 and 21.20 mg/g for BCW200) from the two-compartment first-order model were in agreement with the *q*_e,exp_ gained by the average of the sorption capacities obtained from 96 to 168 h (13.07 mg/g for BC200 and 21.57 mg/g for BCW200) of experimental data, further suggesting that the two-compartment first-order model was suitable for describing the sorption behaviour between OFL and *U.P-*biochar.

**Table 2:** The fitting results of the pseudo-first-order model, pseudo-second-order model and two-compartment first-order model.

Biochars	Pseudo-first-order model			Pseudo-second-order model			Two-compartment first-order model						
	*q*_e,cal_ (mg/g)	*k*_1_ (h^−1^)	*R*^2^	*q*_e,cal_ (mg/g)	*k*_2_ (g/(mg h))	*R*^2^	*q*_e,cal_ (mg/g)	*f*_fast_	*k*_fast_ (h^−1^)	*f*_slow_	*k*_slow_ (h^−1^)	*f*_fast_/ *f*_slow_	*R*^2^
BC200	11.00	4.03	0.708	11.45	0.48	0.812	13.13	0.61	15.93	0.39	0.03	1.55	0.984
BCW200	18.61	1.83	0.764	19.42	0.13	0.865	21.20	0.56	10.28	0.44	0.06	1.25	0.984

It is widely accepted that the solid–liquid sorption process would be dominated by intraparticle diffusion or liquid film diffusion or both combined^48^. Accordingly, the kinetic sorption data were also analysed with the intra particle diffusion and the liquid film diffusion models for identifying which process controlled the rate of sorption for OFL. As illustrated in Fig. 2b and Table 3, the plots of the intraparticle diffusion model did not pass through the origin and the intercepts were positive (7.52 mg/g for BC200 and 11.37 mg/g for BCW200), indicating that the intraparticle diffusion may not have been a primary rate-controlling step in the kinetic process and that sorption occurred mainly on the surface of *U.P-*biochar^48^. Meanwhile, the results of the liquid film diffusion model showed higher *R*^2 ^values (0.930 for BC200 and 0.928 for BCW200) and small intercepts (Fig. 2c and Table 3), conveying that the kinetics of the sorption of OFL on *U.P-*biochar was principally regulated by the liquid film diffusion mechanism^48^.

**Table 3:** The fitting results of the intraparticle diffusion model and liquid-film diffusion model.

Biochars	Intra particle diffusion model			Liquid film diffusion model	
	A mg/g	*k*_a_ mg/(g h^0.5^)	*R*^2^	-*k*_lf_ (h^−1^)	*R*^2^
BC200	7.52	0.52	0.924	0.0267	0.930
BCW200	0.96	11.37	0.841	0.0343	0.928

#### Sorption isotherms

**Figure 3:**
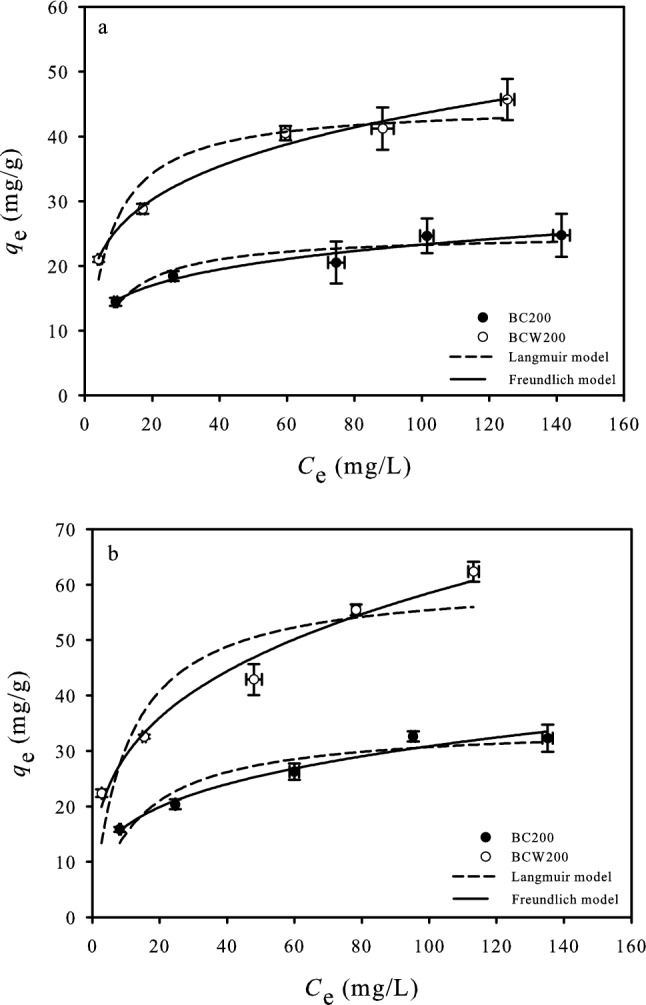
Sorption isotherms of OFL on BC200 and BCW200 at 15 °C (**a**) and 25 °C (**b**). [Conditions] 20 mL of 20–160 mg/L OFL; 0.0150 g of *U.P*-biochar; pH of 7.0 ± 0.2; 7 days. The experiment was conducted in triplicate.

Sorption isotherm describes the correlation between the amount of solute sorbed per unit mass of sorbent and the solute concentration in the solution at equilibrium^49^. The sorption isotherms of OFL on BC200 and BCW200 at 15 °C and 25 °C are presented in Fig. 3. It is shown that the sorption capacity of *U.P-*biochar increased with the increase in the initial concentration of OFL, suggesting that the concentration gradient was the driving force of the sorption^22^. The detailed parameters of the Langmuir and Freundlich models are listed in Table 4. Compared with the Langmuir model, the Freundlich model showed a better representation of the sorption behaviour of OFL on BC200 and BCW200 due to its high correlation coefficients (*R*^2^ > 0.945), implying that the sorption process could be explained by a multilayer sorption mechanism that occurred on a heterogeneous surface^44^. The values of n for the Freundlich isotherm were less than 1, demonstrating that the sorption of OFL on *U.P-*biochar was favourable. The water washing process could decrease the nonlinear sorption degree of OFL for a given temperature with a higher n value, which was attributed to the higher aromaticity and lower pore size of the sorbent that is commonly related to a more nonlinear isotherm.

**Table 4:** Parameters of the sorption isotherms fitted with the Langmuir and Freundlich models.

Temperatures (°C)	Biochars	Langmuir model				Freundlich model				
		*q*_max_ (mg/g)	*K*_L_ (L/mg)	*R*^2^	*R*_L_	n	*K*_f_ (mg g^−1^)/(mg L^−1^)^n^	*R*^2^	*K*_d_ (L/g)	
									*C*_e_ = 5 mg/L	*C*_e_ = 50 mg/L
15	BC200	25.01	0.131	0.880	0.0455	0.196	9.447	0.945	2.590	0.407
	BCW200	44.98	0.159	0.914	0.0378	0.227	15.341	0.989	4.418	0.744
25	BC200	34.71	0.077	0.901	0.0751	0.273	8.768	0.967	2.722	0.511
	BCW200	60.89	0.101	0.811	0.0583	0.301	14.601	0.975	4.743	0.949

The *K*_d_ values of OFL for BC200 and BCW200 were estimated from the Freundlich model at two given equilibration concentrations (*C*_e_ = 5 mg/L and 50 mg/L). The *K*_d_ values decreased with the increase in the concentration of OFL because of the limited high-energy sorption sites on *U.P*-biochar^50^. However, the findings of Huang et al. showed that the *K*_d_ values increased with the initial OFL concentration for the cassava residue-derived biochar prepared at pyrolysis temperatures of 350 °C, 650 °C and 750°C^7^, which was likely due to the principal sorption mechanism (such as micropore filling). The *K*_d_ of BCW200 was significantly higher than that of BC200, suggesting that the water washing process could enhance the sorption affinity of OFL towards *U.P-*biochar. Meanwhile, the *q*_max_ of BC200 and BWC200 were 25.01 mg/g and 44.98 mg/g at 15 °C, 34.71 mg/g and 60.89 mg/g at 25 °C, respectively. Thus, the findings clearly demonstrated that the sorption of OFL on BC200 and BCW200 became more favourable with increasing temperature. Also, all the values of *R*_L_ between 0 and 1 were a sign of the favourable sorption of OFL on *U.P-*biochar, as a value equal to zeroreveals the reaction is irreversible and > 1 is unfavourable^48^. Comparing with previous studies, the sorption capacity of BCW200 were higher than those of most of biochars in the previous reports (Supplementary Information Table S2), suggestting that the *U.P-*biochar might be successfully utilised as an effective sorbent for OFL removal.

#### Thermodynamic analysis

The values of *K*_d_ and *q*_max_ varied with the solution temperature (Table 4), indicating that the temperature played a vital role in the sorption process. Therefore, the thermodynamic parameters for the sorption of OFL onto BC200 and BCW200 were estimated clearly, which could provide in depth information on the inherent energetic changes during the sorption process. It should be realised that the determination of ∆*G*°, ∆*H*°, and ∆*S*° indeed was dependent on the estimate of the *K*_0_. As shown in Table 5, the sorption of OFL on BC200 and BCW200 occurred spontaneously due to the negative values of Δ*G*°^51^. The values of Δ*G*° for a given biochar decreased with increasing temperature, suggesting that the sorption became more favourable at higher temperatures. Moreover, the Δ*G*° values of BCW200 were more negative than those of BC200, which also confirmed that the sorption affinities of OFL on BCW200 were greater than those on BC200. The positive values of ∆*H*° implied that the sorption of OFL on BC200 and BCW200 was endothermic; therefore, the temperature elevation accelerated the sorption process^52^. The positive values of Δ*S*° reflected an increase in the disorder and randomness at the solid–liquid interface during the sorption process^52^.

**Table 5:** The thermodynamic parameters of sorption of OFL by BC200 and BCW200.

Biochars	Temperatures (°C)	ln*K*_0_	Δ*G*° (KJ/mol)	Δ*H*° (KJ/mol)	Δ*S*° (J/(mol *K*))
BC200	15	7.15	− 17.12	10.65	96.42
	25	7.30	− 18.09		
BCW200	15	8.08	− 19.35	19.59	135.23
	25	8.36	− 20.71		

## Conclusions

This study demonstrated that the *U.P-*biochar properties and the sorption characteristics of OFL varied significantly after water washing treatment. The aromaticity and amount of O-containing functional groups of washed *U.P-*biochar were lower than those of unwashed *U.P-*biochar and the water washing treatment reduced the pH_pzc_ of *U.P-*biochar. Furthermore, the washed *U.P-*biochar showed a much higher sorption capacity of OFL than the unwashed *U.P*-biochar. The effects of pyrolysis temperature on the *U.P-*biochar sorption of OFL were significantly dependent on the solution pH due to different sorption mechanisms (e.g. cation exchange, electrostatic attraction, H-bond, and cationic–π and π–π interactions) involved in diverse pH regions. In particular, BC200 and BCW200 exhibited a higher sorption capacity of OFL at neutrality. The fast sorption rate of BC200 was higher than that of BCW200 and the ratio of OFL fast and slow sorption fractions decreased after water washing treatment of *U.P*-biochar. The sorption affinity of BCW200 was greater than that of BC200, and the sorption coefficient increased with lower given equilibration concentration and higher solution temperature. Thermodynamic parameters demonstrated that the sorption of OFL on BC200 and BCW200 was spontaneous and endothermic. It is experimentally concluded that washed *U.P*-biochar can serve as a low cost and effective sorbent for OFL removal from water, but the selection of the optimal pyrolysis temperature should be based on the actual situation.

## Materials and methods

### Chemicals and materials

*U. prolifera*was acquired from the Xiangshan Xuwen Seaweed Development Co., Ltd. in Zhejiang Province, China. OFL (analytical standard, 99.9%) was purchased from Aladdin Inc. (Shanghai, China). All chemicals in the experiment were of analytical grade or better and used without further purification. The selected physicochemical properties of OFL are listed in Supplementary Information Table S1.

### Biochar production and characterisation

The preparation of *U.P*-biochar was detailed in our previous study^53^. The produced biochars are hereafter referred to as BC200, BC300, BC400, BC500 and BC600, where the suffix number represents the pyrolysis temperature; BC0 is the raw *U. prolifera* biomass. The pristine *U.P*-biochars were mixed with deionised water and the solid–liquid ratio was 1:100 (w/v). The mixture was shaken with an incubator shaker (180 rpm, 25 °C ± 1 °C) in the dark for 12 h. Subsequently, the samples were filtered through a 0.45-μm pore size membrane under vacuum for separating the washed *U.P*-biochar. The above process was repeated until the absorption spectra of the filtrate are close to that of deionised water. Finally, the washed *U.P*-biochars were dried in an oven at 105 °C until constant weight and milled to pass through a 100-mesh sieve. The washed *U.P*-biochars were labelled as BCW200, BCW300, BCW400, BCW500 and BCW600.

The elemental C, N, H and O abundances of *U.P*-biochar were measured with an elemental analyser (Vario ELIII, Elementar, Germany). The surface areas and porous structures were determined by N_2_ adsorption at 77 K using a volumetric gas adsorption instrument (ASAP2460, Micromeritics, USA). The FTIR spectra were qualitatively recorded by a Fourier transform infrared spectrometer (Nicolet iS5, Thermo Fisher, USA) in the range of 400–4000 cm^−1^. The pH_pzc_ was measured as described in a previous study^35^.

### Batch sorption experiments

Batch sorption experiments compared the sorption capacities of OFL by the washed and unwashed *U.P*-biochars for exploring the influences of water washing treatment and assessing the optimal pyrolysis temperature and solution pH. The preliminary experiment showed that the initial solution pH would be significantly changed by the addition of *U.P*-biochar and the equilibrium pH was similar for the specific biochar. Thus, the pH of the biochar solution was adjusted before mixing with OFL for better understanding the effect of solution pH on the sorption of OFL by *U.P*-biochar. Briefly, approximately 0.0150 g of *U.P*-biochar was pre-wetted in a 12 mL background solution (0.01 mol/L NaCl and 200 mg/L NaN_3_ as a biocide) and adjusted to the desired pH (3, 5, 7, 9 and 11, the standard deviation of each pH was less than 0.2.) by 1.0 mol/L or 0.1 mol/L HCl and NaOH and then the biochar solution was shaken (180 rpm) in the dark. This process was repeated 2 times a day until the pH was stable. The total volumetric amounts of acid and base applied in all practical solutions were less than 200 µL. After that, the OFL working solutions (50 mg/L) at the predefined pH were also prepared by the background solution. Subsequently, 8 mL of OFL working solution was added to the pretreated *U.P*-biochar samples with an initial OFL concentration of 20 mg/L. The vials were shaken using a shaking incubator (180 rpm, 25 °C ± 1 °C) in the dark for 7 days to achieve sorption equilibrium. Then, the samples were filtered through a 0.45-μm hydrophilic polyethersulfone membrane filter. Finally, the concentration of OFL in the filtrate was analysed by a UV–visible spectrophotometer (Cary50, Varian, USA) at 287 nm. The filtrate was diluted 10 times by distilled water and the pH of all samples was adjusted to 7.0 ± 0.2 before the OFL was measured. The standard curve and regression equation of OFL are shown in Supplementary Information Fig. S3.

The sorption kinetics of OFL by *U.P*-biochar was conducted at a neutral pH of 7.0 ± 0.2. Briefly, 8 mL of OFL working solution (50 mg/L) was mixed with 12 mL of pretreated *U.P*-biochar solution with an initial OFL concentration of 20 mg/L. The mixtures were shaken (180 rpm, 25 °C ± 1 °C) in the dark and the samples were taken at predetermined time intervals to measure the kinetics. To avoid changing the kinetic sorption system’s solid–liquid ratio, triplicate samples under the same conditions were sacrificed at each sampling time point.

The sorption isotherm experiments were also performed using the batch equilibration technique at a solution temperature of 15 °C ± 1 °C and 25 °C ± 1 °C. Briefly, 8 mL of OFL working solutions with different concentrations (50 mg/L to 400 mg/L) were, respectively, added to 12 mL of pretreated *U.P*-biochar solution (pH = 7.0 ± 0.2). The initial concentrations of various OFL ranged from 20 mg/L to 160 mg/L. Subsequently, the samples were shaken for 7 days to achieve sorption balance and the residual concentrations of OFL were measured. Also, blank controls containing only OFL or biochar were established and all the experiments were run in triplicate.

### Data analysis

The Lagergren pseudo-first-ordermodel [Eq. (1)], pseudo-second-order model [Eq. (2)], two-compartment first-order model [Eq. (3)], Weber-Morris Intra particle diffusion model [Eq. (4)] and liquid film diffusion model [Eq. (5)] were devoted to explain the sorption kinetics of OFL by *U.P-*biochar. And both Langmuir model [Eq. (6)] and Freundlich model [Eq. (7)] were applied to portray the sorption isotherms of OFL by *U.P-*biochar. Furthermore, the thermodynamic parameters including the Gibbs free energy change, enthalpy change and entropy change were calculated. The data analysis was conduct by SigmaPlot 10 (Systat Software Inc., CA, USA).

The pseudo-first-order model:

1 $${\text{q}}_{{\rm t}} = {\text{q}}_{{\rm e}}\left(\text{1} - {\text{e}}^{-{\text{k}}_{1}{\text{t}}}\right)$$

The pseudo-second-order model:

2 $${\text{q}}_{{\rm t}} = \frac{{\text{k}}_{2}{{\text{q}}}_{{\rm e}}^{2}{\text{t}}}{\text{1} + {\text{k}}_{2}{{\text{q}}}_{{\rm e}}{\text{t}}}$$

where t (h) is the time for the sorption period; *q*_e_ (mg/g) and *q*_t_ (mg/g) are the sorbed amounts of OFL on *U.P-*biochar at equilibrium and at time *t*, respectively; and *k*_1_ (h^−1^) and *k*_2_ [g/(mg h)] are the rate constants of the pseudo-first-order model and pseudo-second-order model^54^.

Two-compartment first-order model:

3 $${\text{q}}_{{\rm t}} = {\text{q}}_{{\rm e}}{{\text{f}}}_{{\rm fast}}\left(\text{1} - {\text{e}}^{{-{\text{k}}}_{{\rm fast}}{\text{t}}}\right)\text{+}{\text{q}}_{{\rm e}}{{\text{f}}}_{{\rm slow}}\text{(1-}{\text{e}}^{{-{\text{k}}}_{{\rm slow}}{\text{t}}}{)}$$

where *f*_fast_ and *f*_slow_ represent the fractions of the two compartments and *k*_fast_ (h^−1^) and *k*_slow_ (h^−1^) are the rate constants of the two compartments^46^.

Weber-Morris Intraparticle diffusion model:

4 $${\text{q}}_{{\rm t}}\text{=A+}{\text{k}}_{{\rm a}}{{\text{t}}}^{0.5}$$

where A (mg/g) is the intercept of the vertical axis; and *k*_a_ (mg/(g h^0.5^)) is the overall diffusion constant for sorption^54^.

Liquid film diffusion model:

5 $$\ln \left( {1 - {\text{F}}} \right) = - {\text{k}}_{{{\text{lf}}}} {\text{t}}$$

where *k*_lf_ (h^−1^) is the sorption rate constant of the liquid film diffusion model; F is the fractional achievement of equilibrium at time t and it is equal to the ratio of *q*_t_ to *q*_e_^48^.

Langmuir model:

6 $${\text{q}}_{{\rm e}} = \frac{{\text{q}}_{{\rm max}}{{\text{K}}}_{{\rm L}}{{\text{C}}}_{{\rm e}}}{\text{1} + {\text{K}}_{{\rm L}}{{\text{C}}}_{{\rm e}}}$$

Freundlich model:

7 $$q_{{\text{e}}} = K_{{\text{f}}} \cdot C_{{\text{e}}}^{{\text{n}}}$$

where *q*_e_ (mg/g) and *q*_max_ (mg/g) are the sorbed amount of OFL on *U.P-*biochar at equilibrium and the maximum sorption capacity of OFL, respectively; *C*_e_ (mg/L) is the OFL concentration in the aqueous phase at equilibrium; *K*_L_(L/mg) and *K*_f_ (mg g^−1^)/(mg L^−1^)^n^ are the Langmuir and Freundlich sorption coefficients; and n (unitless) is a constant usually used as an indicator of isotherm nonlinearity. The single point sorption coefficient (*K*_d_, L/mg) of OFL on *U.P-*biochar was calculated with the equation *K*_d_ = *q*_e_/*C*_e_ at a given equilibrium concentration^55^.

In addition, the separation factor (*R*_L_) based on the Langmuir model was evaluate by using the [Eq. (8)].

8 $${\text{R}}_{{\rm L}} = \frac{1}{\text{1} + {\text{K}}_{{\rm L}}{{\text{C}}}_{0}}$$

where *C*_0_ is the highest initial OFL concentration (mg/L). This parameter indicates that the isotherm is unfavourable (*R*_L_ > 1), favourable (*R*_L_ < 1), linear (*R*_L_ = 1), or irreversible (*R*_L_ = 0)^48^.

The thermodynamic parameters were analysed by the following [Eqs. (9–10)].

9 $$\Delta {\text{G}}^{^\circ } = - {\text{RT}}\ln {\text{K}}_{0}$$

10 $$\ln \left[ {\frac{{{\text{K}}_{{0{\text{T}}_{2} }} }}{{{\text{K}}_{{0{\text{T}}_{{1}} }} }}} \right] = - \frac{{\Delta {\text{H}}^{^\circ } }}{{\text{R}}}\left[ {\frac{1}{{{\text{T}}_{2} }} - \frac{1}{{{\text{T}}_{1} }}} \right]$$

11 $$\Delta {\text{G}}^{^\circ } = \Delta {\text{H}}^{^\circ } - {\text{T}}\Delta {\text{S}}^{^\circ }$$

where *K*_0_ is the equilibrium partition constant, Δ*G*° (kJ/mol) is the Gibbs free energy change, Δ*H*° (kJ/mol) is the enthalpy change, Δ*S*° (kJ/(mol K) is the standard entropy change, *R* (8.314 J/(mol K)) is the universal gas constant and *T*(K) is the absolute temperature. The thermodynamic constant (*K*_0_; dimensionless) can been valuated by plotting ln (*q*_e_/*C*_e_) versus *C*_e_ and extrapolating *C*_e_ to zero. The value of the intercept is that of ln*K*_0_^56^.

## Supplementary Information

Supplementary Information.

## Data Availability

All datasets generated and/or analyzedduring the current study are included in this published article.
