# Supplementary material for: Influence of water washing treatment on Ulva prolifera-derived biochar properties and sorption characteristics of ofloxacin
Source: Sci Rep. 2021 Jan 19;11:1797. doi: 10.1038/s41598-021-81314-4 (PMC7815725; doi:10.1038/s41598-021-81314-4)
Supplement: Supplementary file 1 — Supplementary Information. [file 41598_2021_81314_MOESM1_ESM.pdf]

Supplementary Information

Influence of water washing treatment on *Ulva prolifera*-derived biochar properties and sorption characteristics of ofloxacin

Chenghu Yang[1,2,a], Shichao Miao[2,a], Tiejun Li[1,*]

[1] Zhejiang Marine Fisheries Research Institute, Key Laboratory of Sustainable Utilization of Technology Research for Fishery Resource of Zhejiang Province, Zhoushan, 316021, PR China

[2] Marine and Fishery institute of Zhejiang Ocean University, Zhoushan 316021, PR China

[*]Corresponding author: Tiejun Li

Address: 28 Tiyu Road, Zhoushan City, Zhejiang Province, China.

E-mail address: litiejun19821204@126.com

Tel: +86-580-2299886

[a] These authors contributed equally to this work.

Submitted to ***Scientific Reports***

Table S1. Partial physicochemical properties of OFL[1].

Antibiotic	C_s^+(×10^3 mg/L)	C_s^0(×10^3 mg/L)	C_s^-(×10^3 mg/L)	logk_{ow}	pK_a
OFL	4.31 ±0.14	2.55 ±0.22	3.67 ±0.15	-0.39	6.10/8.28

Table S2. Sorption capacities of different biochars for OFL in the previous studies.

Biochars	Sorption capacity (mg/g)	References
Cassava residue-derived biochar	2.9	[2]
Chitosan/reed biochar composite	5.0	[3]
Luffa sponge-derived biochar	131.9	[4]
Banana peel-derived biochar	3.33	[5]
Corncob-derived biochar	1.39	
Rice-husk-derived biochar	7.72	[6]
Wood-chip-derived biochar	4.99	
Lake sediment-derived biochar	31.62	[7]
Ulva prolifera-derived biochar	60.89	This work

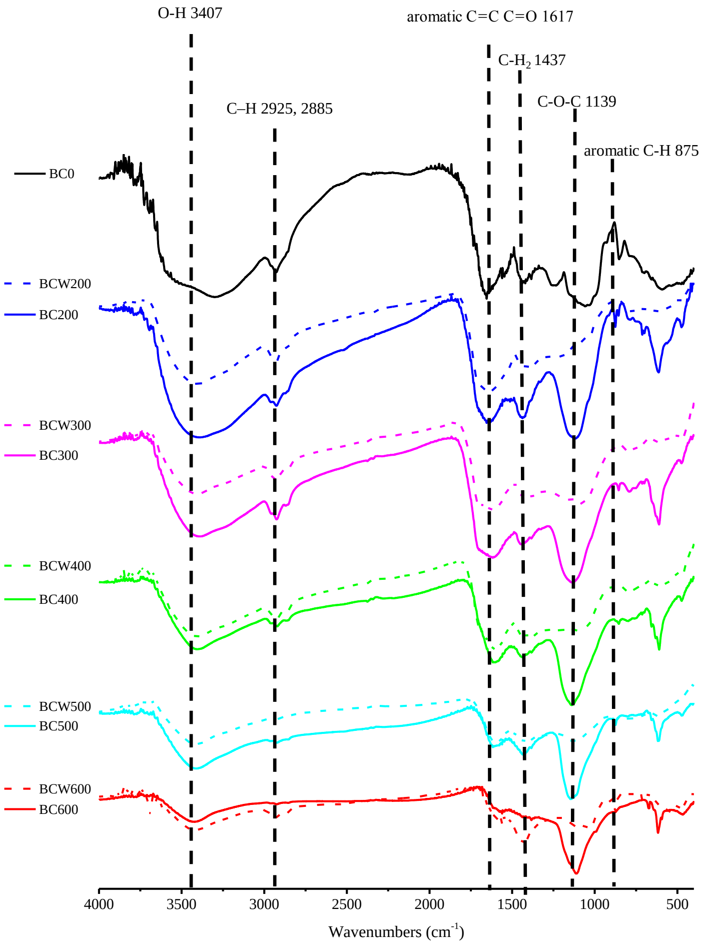

Figure S1. The FTIR spectra of the washed and unwashed *U.P*-biochars.

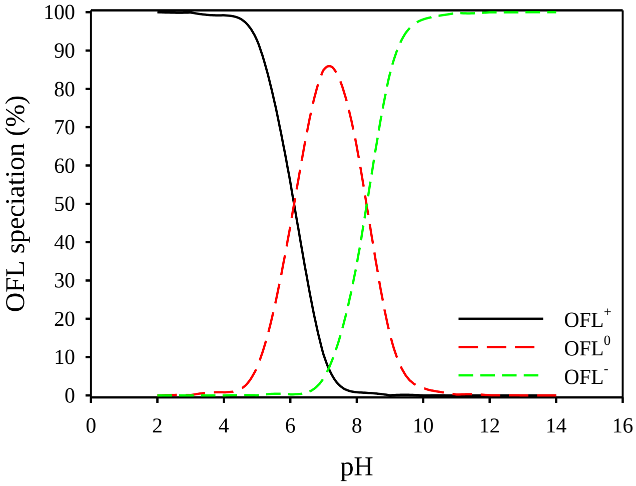

Figure S2. Species distribution of OFL at various pH.

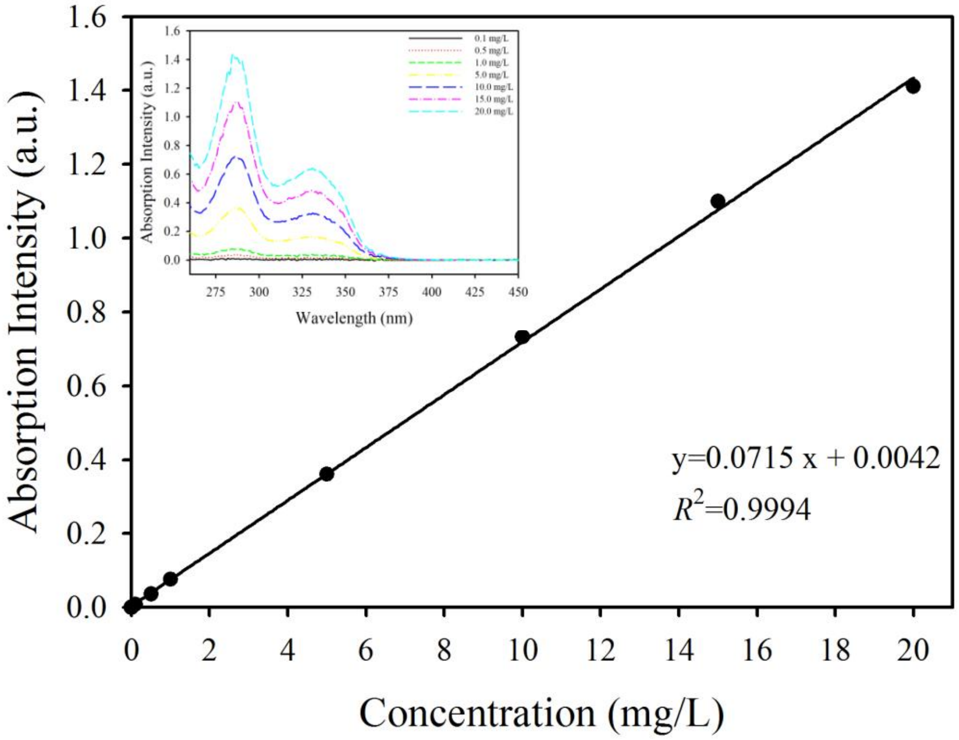

Figure S3. The standard curve and regression equation of OFL.

References

1 Peng, H. *et al.* Adsorption of ofloxacin on carbon nanotubes: Solubility, pH and cosolvent effects. *J. Hazard. Mater.* **211-212**, 342-348 (2012).

2 Huang, P. *et al.* Effects of metal ions and pH on ofloxacin sorption to cassava residue-derived biochar. *Sci. Total Environ.* **616-617**, 1384-1391 (2018).

3 Zhu, C., Lang, Y., Liu, B. & Zhao, H. Ofloxacin adsorption on chitosan/biochar composite: Kinetics, isotherms, and effects of solution chemistry. *Polycyclic Aromat. Compd.* **39**, 287-297 (2019).

4 Kong, Q., He, X., Shu, L. & Miao, M. S. Ofloxacin adsorption by activated carbon derived from luffa sponge: Kinetic, isotherm, and thermodynamic analyses. *Process Saf. Environ. Prot.* **112**, 254-264 (2017).

5 Huang, Q., Wang, P., Zhang, H., Zhang, D. & Yue, Z. Sorption kinetics of ofloxacin by carbonaceous sorbents with different characteristics. *Environ. Chem.* **35**, 651-657 (2016). (in Chinese)

6 Yi, S. *et al.* Removal of levofloxacin from aqueous solution using rice-husk and wood-chip biochars. *Chemosphere* **150**, 694-701 (2016).

7 Wu, M. *et al.* The sorption of organic contaminants on biochars derived from sediments with high organic carbon content. *Chemosphere* **90**, 782-788 (2013).
